# Comparison of Outcomes Between Low-Risk Aortic Valve Replacement Trials and a Surgical Registry

**DOI:** 10.1001/jamanetworkopen.2024.53267

**Published:** 2025-01-06

**Authors:** Makoto Mori, Kayoko Shioda, Christina Waldron, Chenxi Huang, Mario Gaudino, Isaac George, Hiroo Takayama, Arnar Geirsson

**Affiliations:** 1 Division of Cardiac Surgery, Yale School of Medicine, New Haven, Connecticut; 2 Center for Outcomes Research and Evaluation, Yale-New Haven Hospital, New Haven, Connecticut; 3 Department of Global Health, Boston University School of Public Health, Boston, Massachusetts; 4 Center on Emerging Infectious Diseases, Boston University, Boston, Massachusetts; 5 Department of Cardiothoracic Surgery, Weill Cornell Medicine, New York, New York; 6 Division of Cardiothoracic and Vascular Surgery, Columbia University Irving Medical Center, New York, New York

## Abstract

**Importance:**

It remains unknown whether outcomes of the Placement of Aortic Transcatheter Valves 3 (PARTNER 3) and Evolut Low Risk trials are comparable with surgical outcomes in nontrial settings, considering the added risk of concomitant cardiac operations.

**Objective:**

To compare 30-day mortality and stroke incidences of patients in the surgical aortic valve replacement (SAVR) arm of low-risk trials with those of similar patients in the US Society of Thoracic Surgeons Adult Cardiac Surgery Database (STS ACSD).

**Design, Setting, and Participants:**

A cross-sectional sampling study was conducted of adults in the STS ACSD with severe aortic stenosis at low surgical risk for AVR who underwent SAVR during the years low-risk AVR trials (PARTNER 3 and Evolut Low Risk) were enrolling (calendar years 2016-2018). After trial exclusion criteria were applied, 1000 samples were created, each including 1000 patients selected from the STS ACSD according to the likelihood-based selection probabilities to achieve similar STS predicted risk of mortality (PROM) distribution to the trial participants. The distribution of 30-day mortality and stroke rates among these samples were compared with the results from the trials. The analysis was conducted between October 2, 2023, and May 27, 2024.

**Main Outcomes and Measures:**

The main outcomes were 30-day postoperative mortality and stroke.

**Results:**

Among 25 811 patients at low risk undergoing SAVR, the mean (SD) age was 71 (7) years, including 650 220 men (65%). The probability-based sampling yielded a trial-like sample with STS PROM (mean [SD], 1.9% [0.6%]) and concomitant coronary artery bypass graft surgery frequencies of 13%, comparable with the trials. Among the sampled cohorts, the mean (SD) 30-day mortality rate was 1.39% (0.38%) which was not significantly different from the mortality rate in PARTNER 3 (1.1%) (*P* = .83) and Evolut Low Risk (1.3%) (*P* = .65). The stroke rate was 1.25% (0.36%), significantly lower than PARTNER 3 (2.4%) (*P* = .002) and Evolut Low Risk (3.4%) (*P* < .001).

**Conclusions and Relevance:**

In this cross-sectional study, the national samples of low-risk trial-like patients undergoing SAVR during the trial enrollment period had similar 30-day mortality but a lower incidence of stroke compared with the SAVR arm of both low-risk trials. These findings overall suggest that the low-risk trial findings may be generalizable to the broader national SAVR cohort.

## Key Points

**Question:**

Are the short-term outcomes of the surgical aortic valve replacement (SAVR) arm of low-risk aortic valve replacement trials (Placement of Aortic Transcatheter Valves 3 [PARTNER 3] and Evolut Low Risk) generalizable to the clinical practice registry data?

**Findings:**

In a cross-sectional study of 25 811 low-risk trial-like patients in the registry undergoing SAVR, the 30-day mortality rate was 1.4%, comparable with the rate in the low-risk trials. The stroke rate was 1.2%, which was significantly lower than the rate in PARTNER 3 and Evolut Low Risk.

**Meaning:**

The findings of this trial suggest the generalizability of low-risk trial findings.

## Teaser

This cross-sectional study compares the incidence of mortality and stroke among patients undergoing surgical aortic valve replacement in the randomized clinical trial setting with that of similar patients in a registry database.

## Introduction

The Placement of Aortic Transcatheter Valves 3 (PARTNER 3) trial^1^ and Evolut Low Risk trial^2^ reported superior and noninferior death or stroke rate with transcatheter aortic valve replacement (TAVR) compared with surgical aortic valve replacement (SAVR) at 30 days in patients with severe aortic stenosis deemed low risk for AVR. The perioperative divergence between TAVR and SAVR event rates may have a key influence on the long-term outcomes.^3,4^ It remains unknown whether the perioperative SAVR outcomes of the trials were comparable to the outcomes of patients at low risk undergoing SAVR in clinical practice.

Additionally, the SAVR arms of both trials included concomitant surgeries that are known to increase perioperative mortality and stroke. Notable concomitant operations are coronary artery bypass graft (CABG), which was performed in 13% of patients undergoing SAVR in Evolut Low Risk and 14% of those in PARTNER 3, and annular enlargement in 2% of patients in Evolut Low Risk and 5% of those in PARTNER 3. The impact of such concomitant operations on the perioperative outcomes of the SAVR arm is unknown and important to investigate to better contextualize the comparative outcomes reported in the trials.^5,6^

As the trial results were pivotal to the guideline recommendation of TAVR use in the low-risk stratum of patients,^7^ it is important to evaluate whether the SAVR arm trial results incurred expected rates of stroke and mortality compared with the rates in patients with comparable characteristics undergoing SAVR in clinical practice.^8^ Additionally, concordance or discordance of event rates between the trials and the Society of Thoracic Surgeons Adult Cardiac Surgery Database (STS ACSD) in risk-similar patient groups would have important implications toward end point adjudication of future pragmatic trials that increasingly rely on linking multiple data sources. Using the STS ACSD, we aimed to evaluate the likelihood that the 30-day stroke and mortality rates in the trial SAVR arms were comparable to the clinical practice setting outcomes and quantify the potential impact of concomitant operations at frequencies performed in the trial in the outcomes of SAVR.

## Methods

### Data Source and Patients

We used the national STS ACSD to conduct a cross-sectional study of patients at low risk, defined as STS predicted risk of mortality (PROM) less than 4%, which is harmonized with the PARTNER 3 low-risk definition, with severe aortic stenosis who underwent isolated or concomitant SAVR with a bioprosthetic valve between calendar years 2016 and 2018, the years when low-risk trials were enrolling. We applied the trial exclusion criteria to the dataset (eTable 2 in Supplement 1). Key exclusion criteria were patients with a bicuspid aortic valve; moderate or severe aortic, mitral, or tricuspid insufficiency; prior valve operation; in cardiogenic shock with or without mechanical circulatory support; and endocarditis. The STS ACSD captures 97% of all adult cardiac surgery cases performed in the US,^9^ making this dataset the most appropriate to study clinical details of patients undergoing SAVR in the US. The Yale Institutional Review Board and Advarra Institutional Review Board approved the study, and individual consent was waived because of the use of retrospective deidentified data. This study followed the Strengthening the Reporting of Observational Studies in Epidemiology (STROBE) reporting guideline for cross-sectional study. The analysis was conducted between October 2, 2023, and May 27, 2024.

### Variables and Outcomes

Variables were defined per the STS ACSD. Outcomes were (1) 30-day mortality; (2) 30-day stroke rates, which the STS ACSD defines as neurologic deficit caused by malperfusion of the brain lasting greater than 24 hours with neuroimaging or without neuroimaging with clinical determination, or deficits lasting less than 24 hours with neuroimaging confirmation; and (3) a composite of 30-day mortality or stroke rate as a binary end point, counted as an event when the patient experienced either or both mortality or stroke within 30 days from the operation. The STS ACSD codes postoperative stroke during the index hospitalization. Postdischarge stroke was defined as readmission within 30 days of the operation with the reason for readmission documented as stroke. PARTNER 3 used a similar stroke definition up to 90 days, after which the modified Rankin Scale score was used to define disabling vs nondisabling stroke. The Evolut Low Risk trial used a similar stroke definition but reported a 30-day composite stroke or death end point, with the disabling stroke definition determined using the modified Rankin Scale score. The composite end point of any stroke or death was not reported at 30 days in the Evolut Low Risk trial. The STS ACSD does not differentiate disabling vs nondisabling stroke. Comparison of the stroke definitions within the STS ACSD and both trials is summarized in eTable 1 in Supplement 1.

### Statistical Analysis

In the first set of analyses, we created 1000 samples, each including 1000 patients randomly selected with replacement from the STS ACSD, using the likelihood-based selection probabilities to achieve similar frequencies of concomitant CABG and the mean STS PROM in the trial participants. Specifically, a β distribution with the mean (SD) STS PROM of 1.9% (0.6%) (both values from the trials) was used to generate the distribution of the selection probability. According to this β distribution, we assigned the selection probability to each patient based on their STS PROM. The analyzed samples were obtained based on selection probability values for each patient. From the 1000 samples, we analyzed the rates of the 3 outcomes (30-day mortality, stroke, and composite stroke and mortality rates) and compared them with the results from the trials. We performed this sampling approach to assimilate the trial-like patient groups as opposed to the direct comparison of registry and trial participants, as done previously in CABG and percutaneous coronary intervention.^10^

In the second set of analyses, we used the same sampling strategies as the first analysis but only from patients who underwent isolated SAVR. This was performed as a sensitivity analysis to evaluate whether the group consisting only of patients with isolated SAVR at the similar profile would produce outcomes different from those of the trial-like sample including concomitant SAVRs.

In the third set of analyses, the potential impact of concomitant operations on surgical outcomes was evaluated via the following sequence: sampling of the STS ACSD cohort to match the PROM distribution to a mean (SD) of 1.9% (0.6%) along the β distribution and to achieve a concomitant CABG incidence of 13%, and propensity-score matching the patients who underwent CABG plus AVR to those with isolated AVR and substituting the matched CABG plus AVR cohort with the isolated AVR cohort to create a group of patients with isolated SAVR who had the propensity of undergoing concomitant CABG similar to the ones who underwent concomitant CABG. These steps created the sample of patients with isolated AVR, including approximately 13% of patients who could have undergone concomitant CABG, based on the propensity, but did not (eFigure in Supplement 1).

We then characterized the risk profile, patient characteristics, and outcomes of the sampled cohort. We iterated this process 1000 times to derive estimates with CIs. The propensity score was generated by fitting a logistic regression model for the probability of undergoing isolated AVR or CABG plus AVR, with the following covariates: age, sex, body mass index, myocardial infarction, preoperative stroke, carotid artery disease, peripheral vascular disease, preoperative atrial fibrillation, number of diseased coronary vessels, pacemaker, ejection fraction, and preoperative creatinine level. Postoperative stroke rates of patients with and without a history of preoperative atrial fibrillation were compared to infer whether a difference in preoperative atrial fibrillation rates between the samples and the trials may be associated with postoperative stroke rates.

To summarize the rates of the 3 outcomes, means (SDs) of the outcome rates from the 1000 samples are reported. The trial outcome values were compared with the null distribution of the outcome rates of 1000 samples to calculate 1-sided *P* values, with *P* < .05 defined as statistically significant. Because the trials reported continuous variables as means (SDs), we used that measure for continuous variables to make the values comparable. All analyses were conducted with R, version 4.2.3 (R Foundation for Statistical Computing).

## Results

Among 25 811 patients with severe AS at low risk who underwent isolated or concomitant SAVR, the mean (SD) age was 71 (7) years, including 8998 women (35%) and 650 220 men (65%), with the mean (SD) STS PROM of 1.68% (0.81%). Characteristics of patients with SAVR in the national STS ACSD with low surgical risk are summarized in eTable 3 in Supplement 1.

**Figure 1.**
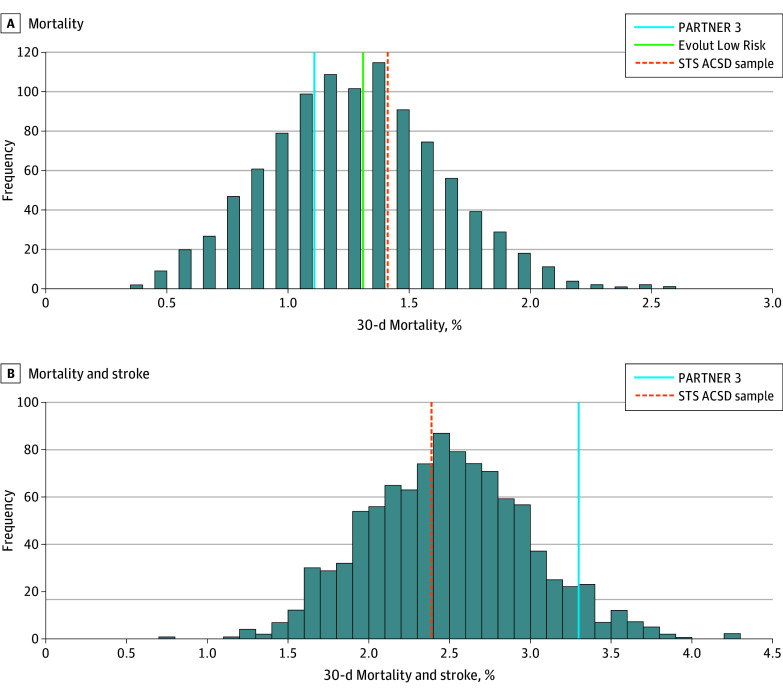
Society of Thoracic Surgeons Adult Cardiac Surgery Database (STS ACSD) Probability Distribution of 30-Day Mortality and Stroke Incidence Distribution of 30-day mortality (A) and stroke (B) outcome rates of 1000 low-risk trial-like samples obtained from the STS ACSD patients undergoing isolated or concomitant surgical aortic valve replacement compared with those in the Placement of Aortic Transcatheter Valves 3 (PARTNER 3) and Evolut Low Risk trials. Mortality and mortality plus stroke rates for both trials fell within the 95% CIs of the samples. Evolut Low Risk only reported the composite of mortality and disabling stroke rate at 30 days and therefore is not displayed.

**Figure 2.**
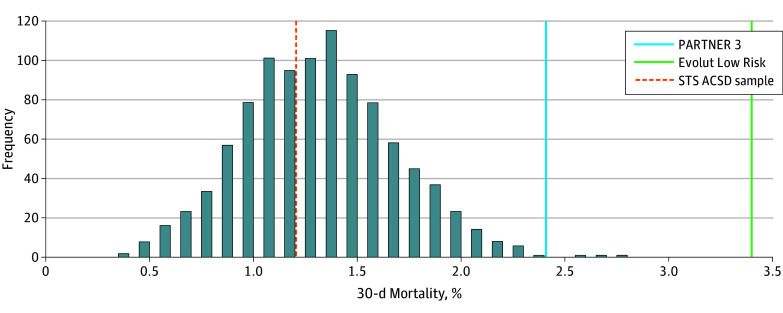
Society of Thoracic Surgeons Adult Cardiac Surgery Database (STS ACSD) Probability Distribution of 30-Day Stroke Incidence Distribution of 30-day stroke rates of 1000 low-risk trial-like samples obtained from the STS ACSD in patients undergoing isolated or concomitant surgical aortic valve replacement compared with those in the Placement of Aortic Transcatheter Valves 3 (PARTNER 3) and Evolut Low Risk trials. Stroke rates of both trials fell above the 95% CIs of the samples.

In the first set of analysis, a total of 1000 samples, each including 1000 patients, yielded STS PROM rates similar to those of PARTNER 3 and Evolut Low Risk (1.9% [0.6%] for the 1000 samples and 1.9% [0.6%] in both trials). Characteristics of the STS ACSD samples and SAVR arms of both trials are summarized in Table 1. The mean 30-day mortality rate of the STS ACSD sample was 1.39% (0.38%). After comparing the mortality rate distribution of 1000 STS ACSD samples with the mortality of the SAVR arms of the low-risk trials, 83.1% of the samples had mortality rates higher than the mortality rate in PARTNER 3 (1.1%) (*P* = .83) and 64.7% had rates higher than those in Evolut Low Risk (1.3%) (*P* = .65), both demonstrating a nonsignificant difference (Figure 1). The mean 30-day stroke rate of the STS ACSD sample was 1.25% (0.36%). Both the PARTNER 3 stroke rate of 2.4% (*P* = .002) and Evolut Low Risk stroke rate of 3.4% (*P* < .001) were significantly higher than STS ACSD samples (Figure 2). The new postoperative pacemaker rate was comparable at 4.5% (0.6%) for the STS ACSD sample, similar to 4.0% for PARTNER 3 (*P* = .81), while statistically significantly lower than 6.1% in Evolut Low Risk (*P* = .006).

**Table 1.** Patient Characteristics of Sampled STS ACSD SAVR Cases and SAVR Arms of Low-Risk Trials

Baseline characteristic	No. (%)		
	STS ACSD sample (n = 1 000 000)	PARTNER 3 (n = 454)	Evolut Low Risk (n = 678)
Age, mean (SD), y	73.1 (7.2)	73.6 (6.1)	73.6 (5.9)
Sex			
Female	349 780 (35.0)	131 (28.9)	229 (33.8)
Male	650 220 (65.0)	323 (71.1)	449 (66.2)
BMI, mean (SD)	31.6 (7.4)	30.3 (5.1)	NA
Race			
White	908 263 (92.9)	409 (90.1)	NA
Other^a^	91 737 (7.1)	45 (9.9)	NA
Coronary artery disease	335 962 (33.6)	127 (28)	NA
Myocardial infarction	107 450 (10.7)	26 (5.8)	33 (4.9)
Diabetes	406 185 (40.6)	137 (30.2)	207 (30.5)
Chronic lung disease			
Mild	116 643 (11.7)	NA	NA
Moderate	45 994 (4.6)	NA	NA
Severe	30 298 (3.0)	NA	NA
COPD	NA	28 (6.2)	117 (18)
Previous stroke	56 601 (5.7)	23 (5.1)	80 (11.8)
Carotid artery disease	116 643 (11.7)	50 (11.3)	NA
Peripheral vascular disease	96 983 (9.7)	33 (7.3)	56 (8.3)
Preoperative atrial fibrillation	77 366 (7.7)	85 (18.8)	98 (14.5)
Creatinine >2 mg/dL	16 906 (1.7)	1 (0.2)	1 (0.1)
Pacemaker	27 851 (2.8)	13 (2.9)	26 (3.8)
LVEF, mean (SD), %	59.6 (9.6)	66.2 (8.6)	61.9 (7.7)
Aortic valve gradient, mean (SD), mm Hg	49.8 (13.7)	48.3 (11.8)	46.6 (12.2)
STS score (predicted mortality), mean (SD), %	1.9 (0.6)	1.9 (0.6)	1.9 (0.7)
STS predicted risk of stroke, mean (SD), %	1.4 (0.6)	NA	NA
Concomitant operations			
CABG	121 724 (12.2)	58 (12.8)	92 (13.6)
Annular enlargement	35 165 (3.5)	21 (4.6)	11 (1.6)
Postoperative TIA	2760 (0.3)	3 (0.7)	5 (0.8)
Postoperative stroke	11 862 (1.2)	11 (2.4)	23 (3.4)
30-d Mortality	13 955 (1.4)	5 (1.1)	9 (1.3)
30-d Mortality or stroke	23 918 (2.4)	15 (3.3)	18 (2.6)
Postoperative atrial fibrillation	356 375 (35.6)	145 (39.5)	240 (35.4)
Postoperative pacemaker	45 331 (4.5)	18 (4.0)	41 (6.1)
Length of stay, median (IQR), d	6 (5-7)	7 (6-8)	NA
30-d Readmission	93 950 (9.4)	29 (6.5)	NA

Abbreviations: BMI, body mass index (calculated as weight in kilograms divided by height in meters squared); CABG, coronary artery bypass graft; COPD, chronic obstructive pulmonary disease; LVEF, left-ventricular ejection fraction; NA, not applicable; PARTNER 3, Placement of Aortic Transcatheter Valves 3; SAVR, surgical aortic valve replacement; STS ACSD, Society of Thoracic Surgeons Adult Cardiac Surgery Database; TIA, transient ischemic attack.

SI conversion: To convert creatinine to micromoles per liter, multiply by 88.4.

^a^ Race was identified according to the STS ACSD definition. Other race category was harmonized with the low-risk trial categorization, including Asian, Black, Native American or Alaskan Native, Pacific Islander, and others who do not fall into these categories.

**Figure 3.**
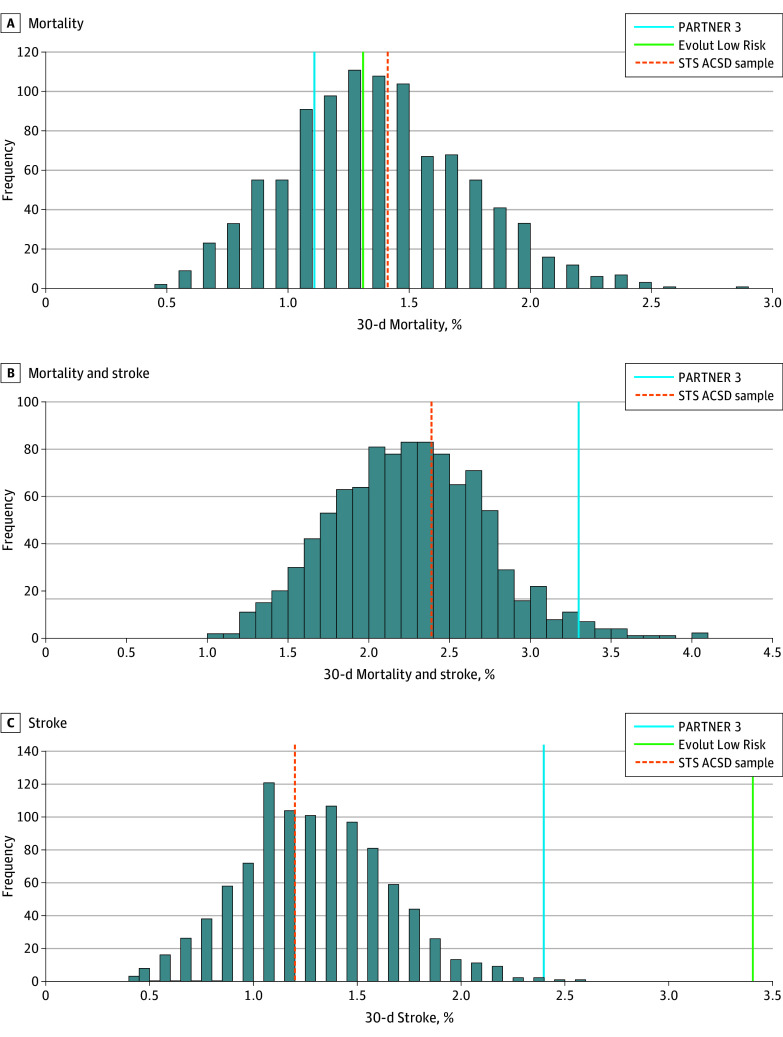
Society of Thoracic Surgeons Adult Cardiac Surgery Database (STS ACSD) Probability Distribution of 30-Day Stroke and Mortality Incidences Among Isolated Surgical Aortic Valve Replacement (SAVR) Distributions of outcome rates of 1000 low-risk trial-like samples obtained from STS ACSD patients undergoing isolated SAVR compared with those in the Placement of Aortic Transcatheter Valves 3 (PARTNER 3) and Evolut Low Risk trials.

In the second set of analyses deriving a separate 1000 samples, each including 1000 patients with isolated SAVR without concomitant operations, the samples yielded an STS PROM of 1.9%. Sample characteristics are summarized in Table 2. The mean 30-day mortality rate of the STS ACSD sample was 1.32% (0.38%). Differences in the mortality rate distribution of 1000 STS ACSD samples compared with the 30-day mortality rate of the SAVR arms of the low-risk trials were nonsignificant (1.1% for PARTNER 3; *P* = .82; and 1.3% for Evolut Low Risk; *P* = .63). The mean 30-day stroke rate of the STS ACSD sample was 1.2% (0.35%). Compared with the STS ACSD sample, the stroke rates were significantly higher in PARTNER 3 (2.4%) (*P* = .004) and Evolut Low Risk (3.4%) (*P* < .001). Specifically, none of the samples had stroke rates higher than those of both trials (Figure 3).

**Table 2.** Patient Characteristics of Sampled Isolated SAVR Cases

Baseline characteristic	STS ACSD sample (n = 1 000 000), No. (%)
Age, mean (SD), y	73.0 (7.1)
Sex	
Female	527 478 (52.7)
Male	472 522 (47.3)
BMI, mean (SD)	31.8 (7.3)
Coronary artery disease	228 991 (22.9)
Myocardial infarction	89 947 (9.0)
Diabetes	395 517 (39.6)
Chronic lung disease	
Mild	115 581 (11.6)
Moderate	42 921 (4.3)
Severe	27 414 (2.7)
Previous stroke	52 631 (5.3)
Carotid disease	114 208 (11.4)
Peripheral vascular disease	88 612 (8.9)
Preoperative atrial fibrillation	51 249 (5.1)
Creatinine >2 mg/dL	15 865 (1.6)
Pacemaker	24 173 (2.4)
LVEF, mean (SD), %	60.1 (9.4)
Aortic valve gradient, mean (SD), mm Hg	50.2 (14.0)
STS score (predicted mortality), mean (SD)	1.9 (0.6)
Postoperative TIA	2793 (0.3)
Postoperative stroke	11 550 (1.2)
30-d Mortality	13 923 (1.4)
30-d Mortality or stroke	23 009 (2.3)
Postoperative atrial fibrillation	360 621 (36.1)
Postoperative pacemaker	44 866 (4.5)
Length of stay, median (IQR), d	6 (5-7)
30-d Readmission	92 705 (9.3)

Abbreviations: BMI, body mass index (calculated as weight in kilograms divided by height in meters squared); LVEF, left-ventricular ejection fraction; SAVR, surgical aortic valve replacement; STS ACSD, Society of Thoracic Surgeons Adult Cardiac Surgery Database; TIA, transient ischemic attack.

SI conversion factor: To convert creatinine to micromoles per liter, multiply by 88.4.

In the third set of analyses deriving a separate 1000 samples, each including 1000 patients with isolated SAVR, based on propensity-score–matched replacement of concomitant cases to yield a sample with a similar prevalence of concomitant operations to the trial while accounting for the fact that the trial STS PROMs were calculated for isolated SAVR, the STS PROM before the matching was 1.9% and after the matching and replacement with isolated SAVR cases was 1.9%. The samples yielded a distribution of mean mortality rates (1.4% [0.38%]) that was not significantly different from low-risk trial outcomes (PARTNER 3, 1.1%; *P* = .82; Evolut Low Risk, 1.3%; *P* = .65), but the stroke rates of both trials were significantly higher (PARTNER 3, 2.4%; *P* = .003; Evolut Low Risk, 3.4%; *P* < .001) compared with the sample distribution of (1.30% [0.35%]). Our comparisons of postoperative stroke and mortality rates by preoperative atrial fibrillation history did not reveal any statistically significant differences between the 2 groups (eTable 4 in Supplement 1).

## Discussion

In this study, we observed that patients who underwent SAVR within the low-risk trials comparing SAVR and TAVR had similar 30-day mortality rates, but a significantly higher 30-day stroke rate compared with patients with a similar risk profile who underwent SAVR in the same time period in the US in a nontrial setting. The finding was not sensitive to multiple key conditions, including sampling exclusively in patients with isolated AVR and propensity-score–based replacement of concomitant cases to isolated AVR cases. Additionally, we observed that concomitant CABG added minimally to the overall risk of the cohort at the frequency performed in the trials, contrary to the prior criticism of the trials that included concomitant cardiac operations known to increase risks.^11^ Together, these findings suggest the generalizability of low-risk trial findings to the broader national SAVR cohort and may have implications in interpreting the long-term follow-up data of such low-risk trials regarding the stroke rates.

Our findings are important for several reasons. First, there remain questions regarding how representative the low-risk trial results are of the clinical practice setting. For example, in the PARTNER 3 trial, 34% of the potential participants who were deemed a reasonable trial candidate by the local heart team to the trial committee were rejected by the trial committee.^1,8^ While patients included in the trial tended to be highly selected and differed in key characteristics from the patients undergoing SAVR in the clinical practice setting,^12^ it remained unknown whether the associations between the patient risk profile (STS PROM) and the risk of outcomes (stroke, mortality) were similar in the trial and the clinical practice setting. By obtaining low-risk trial-like patient groups via a series of probability-based sampling, we observed that the mortality rate was similar given the preoperative risk, but the stroke rate differed between the trials and the national registry. How these findings may translate into the decision to pursue TAVR or SAVR in younger patients, among whom TAVR use is expanding,^13^ also holds important implications.

Second, debate exists on the concomitant cardiac operations potentially biasing the trial results toward worsening the SAVR outcomes, but this potential impact had not been quantified.^14^ We showed that the potential role of the concomitant cases in increasing the perioperative risk is minimal, on the order of a 0.04% increase in the STS PROM for the trial group, and minimal role in the postoperative mortality and stroke incidences. Therefore, while concomitant operations created imbalances in terms of the perioperative risks between TAVR and SAVR, the quantitative role in the overall comparative outcomes of TAVR and SAVR is minimal by this element alone, at least at the relatively low frequency of the concomitant operations performed in the low-risk trials. The contemporary management of concomitant coronary artery disease in the setting of AVR is evolving and moving toward expectant management of stable coronary artery disease at the time of AVR.^15^ The challenge in percutaneous coronary intervention after TAVR must be considered when taking this approach, although the rate of successful percutaneous coronary intervention after TAVR is high in both self- and balloon-expandable valves.^16^

Regarding the divergence of stroke rate between the trials and our study, there are several possible explanations. There may be a difference based on retrospective medical record abstraction in the STS ACSD and the trials’ committee-based adjudication.^17^ While STS data abstraction is standardized, the data harvest is based on retrospective medical record review. A major clinical event, such as stroke, is less likely to be missed even in the review process, nonetheless. Comparison of stroke rates in a randomized clinical trial, adjudicated by the clinical events committee in the Dual Antiplatelet Therapy study, vs those adjudicated by claims data, found concordant event rates (1.9% vs 2.0% at 1 year).^18^ Unlike claims-based data, the STS database is clinical, with abstractors trained specifically for clinical variables. The discordance with the trial event rate using this clinical dataset requires further investigation. This is important as pragmatic trial designs increasingly consider adjunct dataset use to ease the adjudication of trial end points.^19,20^

Third, the higher stroke rates in the trials may be due to the trial participants receiving close attention from multiple parties (clinicians caring for the patient, the local heart team, and the local trial organization), possibly resulting in a lower threshold for obtaining imaging and the detection of subclinical stroke. It may be conceivable that the threshold to trigger neuroimaging may be lower for trial participants for this reason. In fact, the divergence in the rate of stroke in the trials occurred within the median hospital length of stay of 7 days for the SAVR arm and plateaued rapidly following this period. While the stroke risk is the highest in the immediate postoperative period for both SAVR and TAVR, this may suggest that the rate of stroke detection is amplified during the hospital stay, which was longer in the SAVR arm than in the TAVR arm. The proportions of strokes adjudicated via imaging vs clinical assessment alone are not available from the trial publication and may provide additional insights.

The prevalence of atrial fibrillation history was significantly lower in the STS ACSD cohort compared with participants enrolled in the low-risk trials. This discordance is unlikely to be a source of difference in the postoperative stroke incidence, as preoperative atrial fibrillation history was not associated with the risk of postoperative stroke in an earlier STS ACSD analysis.^21^ Our comparisons of postoperative stroke and mortality rates by preoperative atrial fibrillation history also supported that this discrepancy in baseline preoperative atrial fibrillation history likely was not associated with outcome differences.

### Limitations

This study has limitations. It is a comparison of randomized clinical trial results and registry data. While the definitions of 30-day events were similar, the adjudication of stroke in the registry and the trials were different. Underreporting of stroke in the registry is possible depending on the quality of data review by the abstractor. Overreporting is also possible, for example, in a scenario when there is a documentation of suspected stroke in a patient who was subsequently deemed to be without stroke. PARTNER 3 enrolled predominantly in the US, and the STS ACSD likely captures the similar practice pattern, while Evolut Low Risk enrolled internationally, and the SAVR outcome in the STS ACSD population may not be representative of the practice settings of the Evolut Low Risk environment.

The STS PROM mean of 1.9% in both trials was estimated using the model for isolated SAVR, while a small fraction of patients underwent concomitant operations, including CABG, aortic operations, and mitral valve operations. To best simulate this condition given the limitation of the STS PROM calculated as the post hoc performed operations (meaning concomitant AVR CABG risk is calculated based on the AVR plus CABG model), we used propensity-score matching to replace those who underwent concomitant operations and found counterfactual patients similar to those who underwent only isolated AVR.

The TAVR outcomes in trial-like patients undergoing TAVR were not evaluated, and there remains a possibility that TAVR stroke rates were also lower in the nontrial setting. Outcomes data beyond 30 days were not available governed by the nature of the STS ACSD.

## Conclusions

In this cross-sectional study, the national samples of low-risk, trial-like patients undergoing SAVR during the trial enrollment period had similar 30-day mortality but a lower incidence of stroke compared with the SAVR arm of PARTNER 3 and Evolut Low Risk. These findings overall appear to support the low-risk trial findings. While the difference in stroke rates may be due to underrecognition or underreporting of strokes outside of trial settings, these differences should be considered in interpreting the long-term outcome of the trials and in the heart team’s discussion with the patient to determine the best valve replacement option.

## Notes

Supplement 1. **eTable 1.** Comparisons of Stroke Definitions in STS ACSD and Low-Risk Trials **eTable 2.** PARTNER 3 Exclusion Criteria **eTable 3.** Baseline Characteristics of STS ACSD SAVR Patients With Low Surgical Risk **eTable 4.** Comparisons of Patient Characteristics and Outcomes by Preoperative Atrial Fibrillation History **eFigure.** Patient Selection Workflow for Sensitivity Analysis

Supplement 2.
Data Sharing Statement
