# Supplementary material for: Comparison of Outcomes Between Low-Risk Aortic Valve Replacement Trials and a Surgical Registry
Source: JAMA Netw Open. 2025 Jan 6;8(1):e2453267. doi: 10.1001/jamanetworkopen.2024.53267 (PMC11704974; doi:10.1001/jamanetworkopen.2024.53267)
Supplement: Supplement 1. — eTable 1. Comparisons of Stroke Definitions in STS ACSD and Low-Risk Trials eTable 2. PARTNER 3 Exclusion Criteria eTable 3. Baseline Characteristics of STS ACSD SAVR Patients With Low Surgical Risk eTable 4. Comparisons of Patient Characteristics and Outcomes by Preoperative Atrial Fibrillation History eFigure. Patient Selection Workflow for Sensitivity Analysis [file jamanetwopen-e2453267-s001.pdf]

Supplementary Online Content

Mori M, Shioda K, Waldron C, et al. Comparison of outcomes between low-risk aortic valve replacement trials and a surgical registry. *JAMA Netw Open*. 2025;8(1):e2453267. doi:10.1001/jamanetworkopen.2024.53267

eTable 1. Comparisons of Stroke Definitions in STS ACSD and Low-Risk Trials

eTable 2. PARTNER 3 Exclusion Criteria

eTable 3. Baseline Characteristics of STS ACSD SAVR Patients With Low Surgical Risk

eTable 4. Comparisons of Patient Characteristics and Outcomes by Preoperative Atrial Fibrillation History

eFigure. Patient Selection Workflow for Sensitivity Analysis

This supplementary material has been provided by the authors to give readers additional information about their work.

© 2025 Mori M et al. *JAMA Network Open.*

eTable 1. Comparisons of stroke definitions in STS ACSD and low-risk trials

STS ACSD	PARTNER 3	Evolut Low Risk
Acute episode of focal or global neurological dysfunction caused by brain, spinal cord, or retinal vascular injury as a result of hemorrhage or infarction, where the neurological dysfunction lasts for greater than 24 hours.	• Acute episode of a focal or global neurological deficit with at least one of the following: change in level of consciousness, hemiplegia, hemiparesis, numbness, or sensory loss affecting one side of the body, dysphasia or aphasia, hemianopia, amaurosis fugax, or other neurological signs or symptoms consistent with stroke<br>• Duration of a focal or global neurological deficit ≥ 24 h; OR < 24 h, if available neuroimaging documents a new hemorrhage or infarct; OR the neurological deficit results in death.<br>• No other readily identifiable nonstroke cause for the clinical presentation to be determined by or in conjunction with designated neurologist.<br>• Confirmation of the diagnosis by at least a neurology /neurosurgical specialist or non-neurologist physician (if neurologist is not available)	• Acute episode of a focal or global neurological deficit with at least one of the following: change in the level of consciousness, hemiplegia, hemiparesis, numbness, or sensory loss affecting one side of the body, dysphasia or aphasia, hemianopia, amaurosis fugax, or other neurological signs or symptoms consistent with stroke<br>• Duration of a focal or global neurological deficit ≥ 24 h; OR <24 h if available neuroimaging documents a new hemorrhage or infarct; OR the neurological deficit results in death

STS ACSD = Society of Thoracic Surgeons Adult Cardiac Surgery Database

© 2025 Mori M et al. *JAMA Network Open.*

eTable 2: PARTNER 3 exclusion criteria

STS PROM >4%
Infective endocarditis
Cardiogenic shock, emergent/salvage case status
> mild aortic insufficiency
Severe MR/TR, any mitral stenosis
Bicuspid aortic valve
Presence of aortic aneurysm
Robotic-assisted approach
Unplanned concomitant CABG

STS PROM = Society of Thoracic Surgeons predicted risk of mortality; MR = mitral regurgitation; TR= tricuspid regurgitation; CABG = coronary artery bypass graft

© 2025 Mori M et al. *JAMA Network Open.*

eTable 3: Baseline characteristics of STS ACSD SAVR patients with low surgical risk

Baseline characteristics	N=25,811
Age (yr)	71 (7)
Female	8,998 (35%)
Body mass index (kg/m2)	31.1 (7.5)
Race	
White	23,569 (93%)
Black	699 (2.8%)
Asian	240 (1%)
Other	707 (2.8%)
Coronary artery disease	11,349 (44%)
Myocardial infarction	2,542 (9.9%)
Diabetes	9,625 (37%)
Chronic Lung Disease	
Mild	2,524 (9.8%)
Moderate	849 (3.3%)
Severe	511 (2%)
COPD	-
Previous stroke	1,234 (4.8%)
Carotid Disease	2,866 (11%)
Peripheral vacular disease	1,901 (7.4%)
Pre-operative atrial fibrillation	1,506 (5.8%)
Creatinine > 2 mg/dL	263 (1%)
Pacemaker	498 (1.9%)
LVEF (%)	60 (9)
Aortic valve gradient (mmHg)	50 (13)
STS score (predicted mortality)	1.7 (0.8)
STS predicted risk of stroke	1.4 (0.6)

© 2025 Mori M et al. *JAMA Network Open.*

Baseline characteristics	N=25,811
Concomitant Operations	
Concomitant CABG	7,697 (30%)
Annular Enlargement	931 (3.6%)
Post-Operative TIA	85 (0.3%)
Post-Operative Stroke	284 (1.1%)
30-Day Mortality	288 (1.1%)
30-day Mortality or Stroke	561 (2.2%)
Post-Operative atrial fibrillation	9,057 (35%)
Length of Stay	7 (5)
30-day readmission	2,313 (9%)

STS ACSD = Society of Thoracic Surgeons Adult Cardiac Surgery Database; COPD = chronic obstructive pulmonary disease; CABG = coronary artery bypass graft; TIA = transient ischemic attack; LVEF = left ventricular ejection fraction. Continuous variables are displayed as mean (standard deviation).

© 2025 Mori M et al. *JAMA Network Open.*

eTable 4: Comparisons of patient characteristics and outcomes by preoperative atrial fibrillation history

Variables	No preoperative afib (N = 24,305)	Preoperative atrial fibrillation (N = 1,506)	P
Baseline characteristics			
Age (yr)	71.3 (7.5)	72.6 (6.7)	<0.001
Female	8,607 (35.4%)	391 (26.0%)	<0.001
Body mass index (kg/m2)	31.1 (7.6)	31.5 (5.9)	<0.001
STS score (%)	1.7 (0.8)	2.0 (0.9)	<0.001
Coronary artery disease	10,713 (44.1%)	636 (42.2%)	0.2
Myocardial infarction	2,355 (9.7%)	187 (12.4%)	<0.001
Diabetes	9,069 (37.3%)	556 (36.9%)	0.7
Chronic Lung Disease			0.006
Mild	2,360 (9.7%)	164 (10.9%)	
Moderate	797 (3.3%)	52 (3.5%)	
Severe	466 (1.9%)	45 (3.0%)	
Previous stroke	1,131 (4.7%)	103 (6.8%)	<0.001
Carotid Disease	2,670 (11.0%)	196 (13.0%)	0.015
Peripheral vascular disease	1,770 (7.3%)	131 (8.7%)	0.041
Creatinine > 2 mg/dL	252 (1.0%)	11 (0.7%)	0.3
Pacemaker	417 (1.7%)	81 (5.4%)	<0.001
LVEF (%)	60.2 (8.9)	59.0 (9.3)	<0.001
Aortic valve gradient (mmHg)	49.9 (13.5)	47.6 (12.4)	<0.001
Concomitant CABG	7,301 (30.0%)	396 (26.3%)	0.002
Annular enlargement	905 (3.7%)	26 (1.7%)	<0.001

© 2025 Mori M et al. *JAMA Network Open.*

Variables	No preoperative afib (N = 24,305)	Preoperative atrial fibrillation (N = 1,506)	P
Outcomes			
Post-Operative TIA	78 (0.3%)	7 (0.5%)	0.3
Post-Operative Stroke	268 (1.1%)	16 (1.1%)	0.9
30-Day Mortality	270 (1.1%)	18 (1.2%)	0.8
Post-Operative atrial fibrillation	8,685 (35.7%)	372 (24.7%)	<0.001
Length of Stay (day)	7.2 (4.9)	8.2 (5.4)	<0.001
30-day readmission	2,138 (8.8%)	175 (11.6%)	<0.001
30-day Mortality or Stroke	527 (2.2%)	34 (2.3%)	0.8

AFib = atrial fibrillation; STS = Society of Thoracic Surgeons; CABG = coronary artery bypass graft; TIA = transient ischemic attack; LVEF = left ventricular ejection fraction.

© 2025 Mori M et al. *JAMA Network Open.*

eFigure: Patient selection workflow for sensitivity analysis

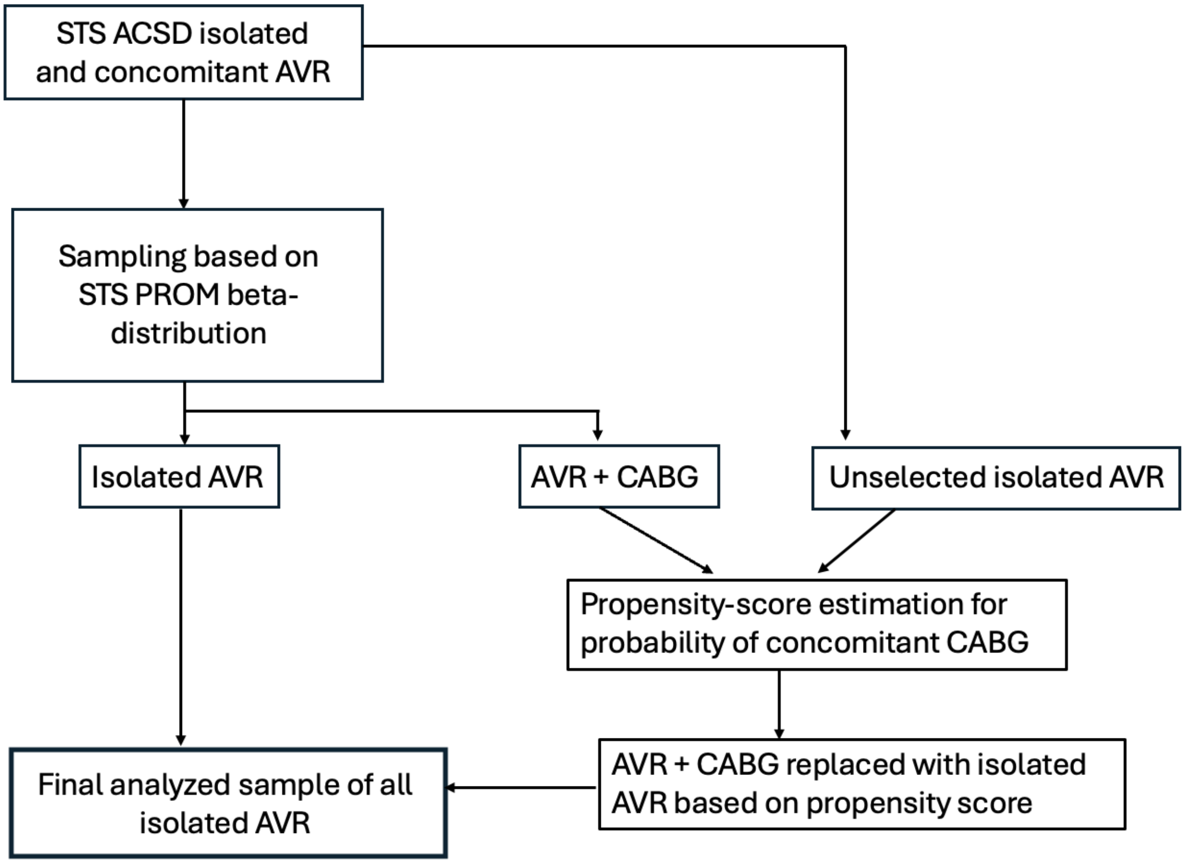

Figure outlines the workflow of identifying isolated aortic valve replacement (AVR) samples based on propensity-score based replacement. STS ACSD = Society of Thoracic Surgeons Adult Cardiac Surgery Database, CABG = coronary artery bypass graft, PROM = predicted risk of mortality.

© 2025 Mori M et al. *JAMA Network Open.*
