# Supplementary material for: Comparison of Outcomes Between Low-Risk Aortic Valve Replacement Trials and a Surgical Registry
Source: JAMA Netw Open. 2025 Jan 6;8(1):e2453267. doi: 10.1001/jamanetworkopen.2024.53267 (PMC11704974; doi:10.1001/jamanetworkopen.2024.53267)
Supplement: Supplement 2. — Data Sharing Statement [file jamanetwopen-e2453267-s002.pdf]

Data Sharing Statement

Mori. Comparison of Outcomes Between Low-Risk Aortic Valve Replacement Trials and a Surgical Registry. *JAMA Netw Open*. Published January 06, 2025.
doi:10.1001/jamanetworkopen.2024.53267

Data
Data available: No

Additional Information
Explanation for why data not available: The STS ACSD data is only made available via the approval of the STS Research Analytic Center.
